# Predicting rice diseases using advanced technologies at different scales: present status and future perspectives

**DOI:** 10.1007/s42994-023-00126-4

**Published:** 2023-11-29

**Authors:** Ruyue Li, Sishi Chen, Haruna Matsumoto, Mostafa Gouda, Yusufjon Gafforov, Mengcen Wang, Yufei Liu

**Affiliations:** 1 College of Biosystems Engineering and Food Science, Zhejiang University, Hangzhou, 310058 China; 2 College of Environmental and Resource Sciences, Zhejiang University, Hangzhou, 310058 China; 3 State Key Laboratory of Rice Biology, and Ministry of Agricultural and Rural Affairs Laboratory of Molecular Biology of Crop Pathogens and Insects, Zhejiang University, Hangzhou, 310058 China; 4 Department of Nutrition and Food Science, National Research Centre, Giza, 12622 Egypt; 5 Central Asian Center for Development Studies, New Uzbekistan University, Tashkent, 100000 Uzbekistan; 6 Global Education Program for AgriScience Frontiers, Graduate School of Agriculture, Hokkaido University, Sapporo, 060-8589 Japan

**Keywords:** Artificial intelligence, Rice disease, Model algorithms, Imaging technology, Plant–pathogen interactions, High-throughput data

## Abstract

The past few years have witnessed significant progress in emerging disease detection techniques for accurately and rapidly tracking rice diseases and predicting potential solutions. In this review we focus on image processing techniques using machine learning (ML) and deep learning (DL) models related to multi-scale rice diseases. Furthermore, we summarize applications of different detection techniques, including genomic, physiological, and biochemical approaches. In addition, we also present the state-of-the-art in contemporary optical sensing applications of pathogen–plant interaction phenotypes. This review serves as a valuable resource for researchers seeking effective solutions to address the challenges of high-throughput data and model recognition for early detection of issues affecting rice crops through ML and DL models.

## Introduction

Rice (*Oryza sativa L*.) is globally recognized as the primary staple food (FAO 2023, Matsumoto et al. 2022). However, its global production is severely threatened by plant diseases, endangering food security in many Asian, African, and European countries (Liu et al. 2023a; Wang et al. 2022; Zhan et al. 2023). Thus, early detection and identification of rice infections are crucial to preventing crop damage and improving yield quality and quantity. Rice disease detection has long been a significant challenge in plant disease management, and there is a pressing need to develop accurate and efficient methods for this purpose within the realm of agriculture.

The conventional method for in situ detection of rice diseases relies on the observations of experienced farmers. While convenient, this approach necessitates highly skilled inspectors to identify the phenotypic expression of different diseases. Alternatively, biochemical technologies offer more precise means of obtaining crop disease information by analyzing susceptible rice tissues based on their chemical and genomic codes (Jansen et al. 2011; Patel et al. 2023). However, these methods are time-consuming, expensive, reliant on laboratories, and require skilled professionals, rendering them unaffordable for most farmers (Wani et al. 2022). Addressing the limitations of these approaches calls for the development of a more accurate and rapid in situ methods for identifying rice diseases and controlling their spread.

**Fig. 1:**
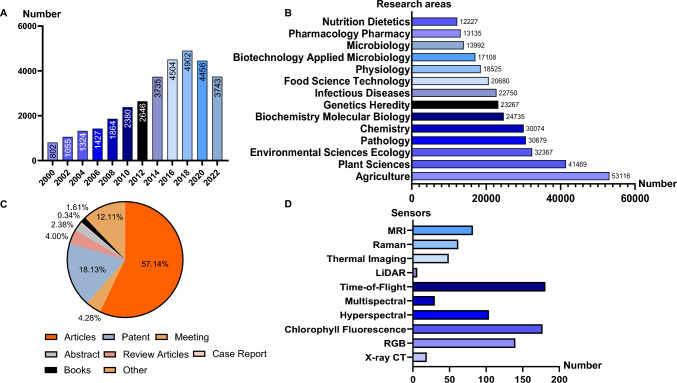
Summary of optical sensing-based phenotyping (OSP) statistics for rice disease from 2000 to 2022. **A** The number of annual publications related to rice disease. **B** The research areas of cereal crop phenotyping publications. **C** The types of publications related to rice disease. **D** The most-mentioned sensors in publications related to cereal crop phenotyping. Note: the statistical data were derived from the Web of Science database (Clarivate Analytics, USA). Publications from 2000 to 2022 were searched with the keywords “X-ray CT” or “RGB” or “chlorophyll fluorescence” or “hyperspectral” or “multispectral” or “ToF” or “LiDAR” or “thermal imaging” or “Raman” and “MRI”. Please note that the final count for “RGB” includes “RGB,” “digital camera”, “digital imaging”, and “visible light imaging” since all of these pertain to the same type of sensor

The demand for advanced technologies in rice disease detection has significantly increased from 2000 to 2020, as evidenced by the surge in related research and publications (Fig. 1).

Optical sensing-based phenotyping (OSP) has become a common approach in nondestructive rice disease detection, with methods like charge-coupled device (CCD) cameras being employed to analyze various features of rice diseases, such as color (Shrivastava and Pradhan 2021), shape (Lu et al. 2022), texture (Ahmad et al. 2021), and spectral reflectance (Tian et al. 2021). However, these technologies have limitations in handling large sample sizes, limited extraction features, inaccurate segmentation, and noise suppression, affecting their accuracy in complex backgrounds and unknown samples (Hamuda et al. 2017).

In recent years, the advent of nondestructive detection technologies has led to the application of new methods for identifying rice seed variety and vigor, including near-infrared (NIR) spectroscopy (Fabiyi et al. 2020), nuclear magnetic resonance (NMR) spectroscopy (Song et al. 2018), Fourier-transform infrared (FTIR) spectroscopy (Kusumaningrum et al. 2018), Raman spectroscopy (Ambrose et al. 2016), terahertz spectroscopy (THz) (Wei et al. 2016), and X-ray imaging (Costa et al. 2014; Ramakrishna 2023). Following the unprecedented progress achieved in computer and electronic technologies, machine learning (ML) and deep learning (DL) have significantly improved image analysis techniques and expedited large data processing (Mahlein et al. 2018; Singh et al. 2018). These methods can provide multidimensional information from rice crop images, including color, near-infrared spectra, three-dimensional (3D), and thermal radiation (Sun et al. 2020). ML and DL have proven effective in plant disease detection through images compared to traditional methods (Gill et al. 2022; Hai et al. 2022), making them invaluable for predicting complex and uncertain rice infections. These techniques hold great promise and widespread application in the realm of rice disease detection.

**Fig. 2:**
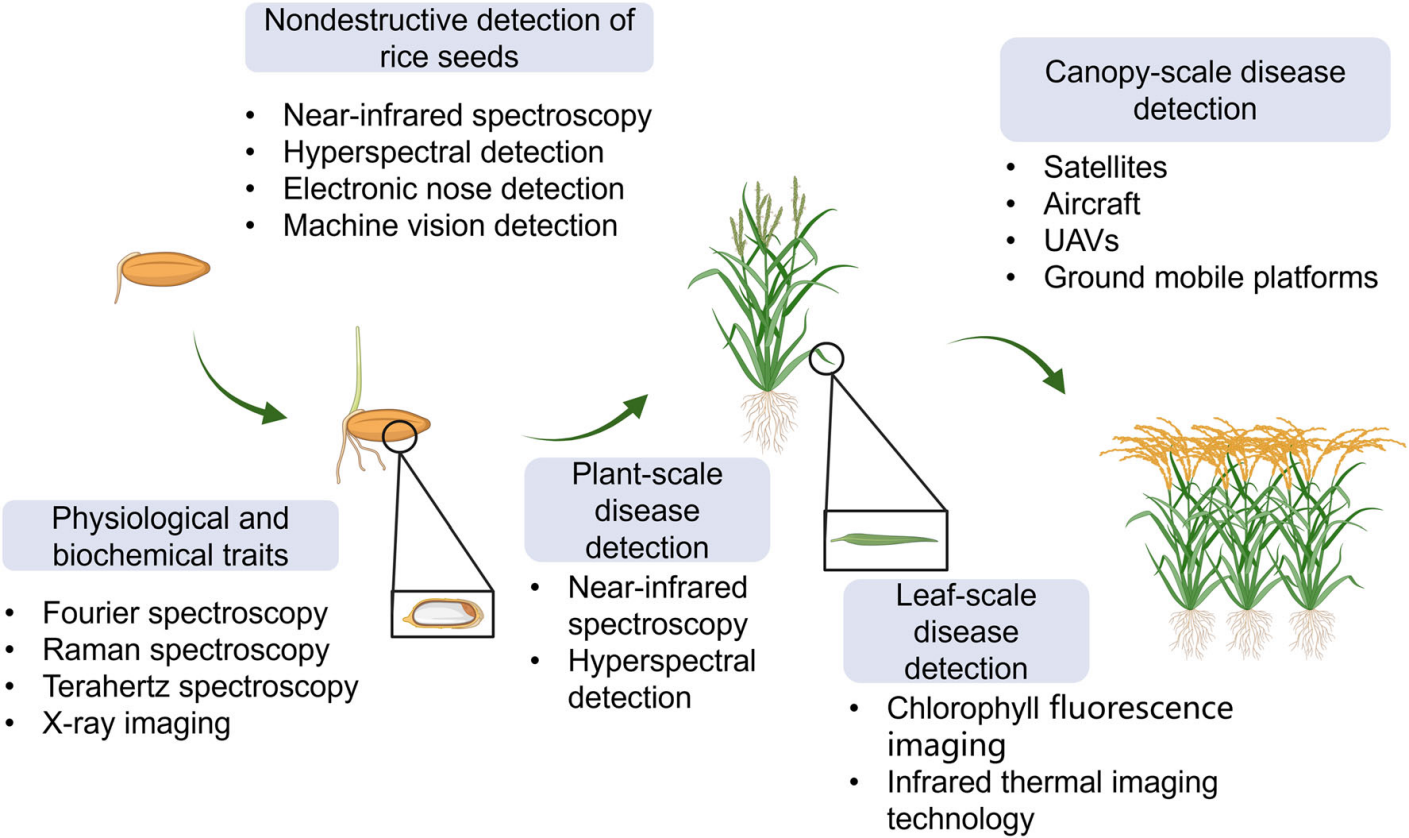
Major rice disease detection methods by advanced technology at different scales (Created with BioRender.com)

This review summarizes the recent major findings of ML and DL in rice disease detection, at multiple scales (Matthews and Marshall-Colón 2021), spanning gene, seed, seedling, and canopy scales. We discuss four key areas: nondestructive seed detection, crop phenotype identification, disease identification using rice physiological indices, and ML and DL applied to plant–microbe interactions (Fig. 2). Our review also underscores the challenges and future perspectives in the field of plant disease detection.

## Nondestructive detection of rice seeds

Rice seeds serve as the backbone of the rice industry (Li et al. 2022a), and selecting high-quality seeds with robust varieties and vitality is crucial for ensuring optimal rice production (Matsumoto et al. 2021). For instance, bacterial diseases in rice have emerged as a significant concern in major rice-producing regions due to their numerous means of transmission, swift outbreaks, and frequent re-infestations (Jung et al. 2018). Moreover, these issues continue to grow annually, posing a serious threat to the safety of rice production. Common rice diseases include rice blast, bakanae disease, bacterial blight, and bacterial leaf streak (Wang et al. 2018; Xu and Chen 2011). The heterogeneity in rice seed genetics and vitality significantly influences their nutrition and disease resistance. However, challenges like biological hybridization during breeding, mechanical processing during harvesting, and illegal trading in the market hinder the promotion of high-quality rice seeds. Traditional detection methods fall short of the requirements for rice seed detection. Hence, various advanced technologies have emerged to enable rapid, non-destructive, and targeted identification of rice seed variety, vitality, and pathogenic microorganisms.

## Seed disease detection

It is now understood that many pathogens can spread, via seeds, through spores or mycelium and become the primary source of field infections (Fan et al. 2019a; Marcel et al. 2010). Distinguishing pathogen-infected seeds from healthy ones through visual observation is challenging. Spectral imaging offers a nondestructive method for diagnosing diseased seeds. In this respect, a recent study (Baek et al. 2019) used various ML models to characterize visible near-infrared spectra bands, enabling the differentiation of infected rice seeds with bacterial cereal blight from healthy ones. Meanwhile, Zhang et al. (2020) employed hyperspectral imaging (HSI) combined with DL to classify rice seed vigor classes at various frost levels. When using spectral preprocessing and feature extraction algorithms, the accuracy of the deep forest (DF) model reached 99.33%, achieving precise classification of rice seeds with different frost levels. Optical instruments, while not inexpensive, have gained recognition for their high accuracy and efficiency.

To address the cost of hyperspectral imaging technology, scholars have developed feature filtering processes to select the optimal features, corresponding to chlorophyll, anthocyanin, fat, and water content in seeds (Chadha 2021; Cheng and Ying 2004; Cheng et al. 2006). This filtering process has the potential for use in the development of cost-effective narrow-channel sensors. Weng et al. (2020) designed a low-cost multispectral imaging system to detect the disease status of rice seeds. Based on spectral single-band features, the least squares support vector machine (LS-SVM) model achieved over 90.3% accuracy in detecting different rice strains and varieties. These advances in spectral methods promise increased efficiency and accuracy in detection and offer valuable guidance for germplasm resource breeding and storage.

## Seed variety identification and viability detection

Cultivating high-quality rice germplasm resources relies on identifying seed varieties and assessing their vitality. Several key factors contribute to predicting viability of rice seed varieties. Germination rate, measured by the percentage of seeds successfully germinating under controlled conditions, is a major determining factor. Additionally, seed moisture content (optimal range: 12–14%) and post-germination growth rates, influenced by stem and root length, weight, and biomass, play essential roles (Zhang et al. 2005). The electrical conductivity of the seed membrane can negatively impact viability by damaging the membrane (Duan et al. 2010). Physical attributes, including color, shape, and weight, serve as indicators of viability. Taken together, these criteria offer a comprehensive assessment of seed viability, aiding in predicting successful cultivation (Wang 2019).

Hyperspectral imaging represents a safer and more cost-effective technique for identifying rice seed varieties compared to other methods like X-ray imaging and magnetic resonance imaging (MRI). It has gained significant popularity in recent years for the quality and variety detection of seeds for various crop (Chu et al. 2022). Non-destructive identification methods for rice seed varieties include computer vision techniques, near-infrared spectroscopy, and hyperspectral imaging. Different rice varieties exhibit unique genetic expressions that manifest in specific external characteristics, such as color, texture, and shape. These attributes can be captured using computer vision techniques to differentiate between seed varieties.

In addition, differences in organic matter content within each variety, such as starch and protein, can be observed in the obtained spectra. NIR spectroscopy and hyperspectral imaging effectively identify seed varieties. For instance, Ansari et al. (2021) used an RGB camera to capture images of three rice seed varieties and extracted shape, texture, and color features. Their support vector machine (SVM) model achieved a classification accuracy of 93.9%, outperforming other classification models. Methods like partial least squares discriminant analysis (PLS-DA), SVM, and K-nearest neighbor (KNN) have proven effective in spectral discrimination, differentiating rice seed varieties.

Joshi et al. (2021) proposed a DL-assisted optical coherence tomography (OCT) technique for subsurface imaging to differentiate between various seed species. After extensive training, the network demonstrated excellent accuracy with test datasets. These techniques can accurately classify seed varieties, even those with morphological similarities, helping to remove variety duplication and assess seed purity. Additionally, non-destructive methods for seed viability detection include oxygen sensor technology, infrared thermography, near-infrared spectroscopy, and hyperspectral imaging. Seeds undergo metabolism during storage, consuming oxygen, protein, and fatty substances, while emitting heat. The metabolic rate varies among vigorous seeds, affecting the amount of oxygen consumed and heat generated. Oxygen sensor technology can efficiently determine seed vigor by detecting oxygen consumption (Rolletschek et al. 2009; Zhao et al. 2013), whereas infrared thermography detects seed vigor by measuring heat generation (Men et al. 2017). For instance, Fang et al. (2016) used infrared thermal imaging to capture images of rice seeds with different vigor levels and established a generalized regression neural network (GRNN) model, achieving a correlation coefficient of 0.9003. Fan et al. (2019b) employed NIR spectroscopy to obtain spectral data for two rice seed types, yielding PLS-DA models with an accuracy of 91.67% for classifying these seeds. The above methods provide the foothold for rapid, non-destructive measurements of rice seed vitality on an industrial or large-scale level. They enhance detection efficiency and accuracy, while providing valuable guidance for germplasm resource breeding and storage.

Although nondestructive seed testing methods offer rapid, cost-effective, pollution-free, repeatable, and easily measurable benefits, there are still challenges to overcome in this research stage. For instance, NIR spectroscopy must address interference from water, temperature, and sample variations. Hyperspectral imaging techniques require the selection of characteristic wavelengths and noise reduction.

## Multi-scale rice disease detection

Leaves serve as essential organs for nutrient production in plants, playing a critical role in photosynthesis and respiration (Krishnamurthy et al. 2015; Zhan et al. 2022). Generally, leaves are the first site for identifying plant diseases, as most disease symptoms initially manifest on leaves (Ebrahimi et al. 2017; García et al. 2017). Detecting rice leaf diseases primarily relies on human experience and symptom comparison or chemical detection techniques. Changes in leaf appearance, such as yellowing, browning, curling, wilting, or the presence of spots or stripes, may indicate disease. Lesions or damaged areas on leaves often signify disease presence, with their size, shape, color, and pattern assisting in disease identification. Traditional laboratory testing plays a pivotal role in identifying diseases and determining the type of pathogens affecting rice leaves. In our technologically advanced era, tools, such as drone imaging and spectral analysis, offer the potential to detect early signs of disease, even before they become visible to the naked eye. Collectively, these criteria form a robust system for predicting rice leaf diseases. Traditional methods struggle to accurately, instantly, and easily identify pests and diseases, affecting early pest and disease control, which can decrease rice yields.

Researchers have extensively examined plant disease phenotypes, at the leaf and other scales, using spectral imaging technology and ML algorithms. Most studies on plant disease phenotypes have employed RGB imaging technology, in combination with image processing algorithms, to distinguish and diagnose plant disease species and disease status. In addition to plant disease phenotypes at the leaf scale, there are unique phenotype characteristics at larger scales, such as the canopy.

## Rice leaf disease discrimination by model algorithm combined techniques

The past few years have witnessed a burgeoning interest in research on image recognition and employed specific classifiers to categorize images as healthy or diseased (Jiang et al. 2020; Singh et al. 2021; Zhao et al. 2022). Over the past decades, popular disease identification techniques included K-nearest neighbors (KNN) (Guettari et al. 2016), SVM (Deepa 2017), Fisher Linear Discriminant (FLD) (Ramezani and Ghaemmaghami 2010), ANN (Sheikhan et al. 2012), and Random Forest (Kodovsky et al. 2012). Furthermore, the success of classical methods in disease recognition have relied on lesion segmentation and manually engineered features, with algorithms like scale-invariant feature transform (SIFT), Gabor transform, seven invariant moments, global–local singular values, and sparse representation proving effective (Guo et al. 2007; Zhang and Wang 2016).

The performance of disease recognition algorithms hinges on numerous variables, including preprocessing and segmentation techniques, feature extraction methods, and the choice of a learning algorithm for classification modeling. Under complex background conditions, many methods struggle to effectively segment the plant and corresponding lesion image from the background, leading to unreliable disease recognition outcomes. As a result, automatic recognition of plant disease images remains challenging due to the complexity of disease images. DL techniques, particularly convolutional neural networks (CNNs), have recently gained popularity to overcome some of these challenges.

In this context, Albattah et al. (2022) enhanced the central network of the target recognition model and identified rice leaf diseases by substituting the backbone network. This approach reduced the number of candidate frames during target recognition, accelerating model inference times. Accuracy and recall rates were approximately 99% during validation tests using publicly available datasets. In DL-based plant disease identification research, model application is crucial in promoting intelligent crop disease management. With the development of Internet of Things (IoT)-based crop monitoring sensor networks and the need for IoT-based intelligent early warning systems for plant disease recognition, many researchers have integrated their own DL algorithm models to create expert systems for plant leaf disease recognition (Jamal and Judith 2023). These systems can accurately and effectively identify plant leaf diseases in real-time and offer comprehensive prevention and control recommendations.

## Canopy-scale disease detection

In crop plants, the canopy, which is responsible for photosynthesis, plays a crucial role in determining the efficient utilization of light energy and photosynthetic nitrogen use efficiency. Remote sensing technologies, using satellites, aircraft, unmanned aerial vehicles (UAVs), and ground mobile platforms, have become valuable tools for efficient disease identification and early diagnosis within plant populations, particularly at the canopy scale (Li et al. 2022b). The primary platforms for monitoring canopy health include satellites, ground-based platforms, and UAVs. These platforms offer different advantages for monitoring rice crop disease at various scales.

Satellite-based platforms are ideal for large-scale monitoring due to their capacity to capture extensive area data at fixed intervals. Advances in satellite technology have improved spatial and temporal resolution, enhancing the accuracy of models based on satellite data. However, challenges like regression cycles, weather interference, and cloud cover can make continuous data acquisition challenging for early disease monitoring.

The UAV platform, situated between ground and satellite scales, offers flexible and cost-effective data acquisition, compensating for satellite platform limitations (Ge et al. 2023; Liu et al. 2023b). Flying at a certain altitude allows wide-range image acquisition, ideal for medium-scale plots like farms and experimental fields. However, canopy-scale imagery poses challenges due to complex backgrounds, textures, occlusions, and reflections, making traditional image processing and ML methods less effective in identifying and detecting issues.

By comparison, DL techniques, with their deep layers for feature abstraction, excel in handling complex models and are increasingly applied in rice canopy disease detection (Azadbakht et al. 2019). With the rapid development of DL and UAV platforms, Wang et al. (2018) utilized a UAV remote sensing platform to develop an Adaboost white spike classifier based on Harr-like features extracted from RGB images of rice spikes, achieving 93.62% accurate recognition of white spikes in rice diseases. Complementing the UAV platform, ground platforms, and portable instruments provide more comprehensive and real-time data, offering enhanced accuracy (Wang et al. 2018). The rapid development of hyperspectral remote sensing technology and the advancements in UAV and satellite scale disease remote sensing suggest that large-scale air-ground disease monitoring will gradually become achievable.

## Advanced physiological and biochemical disease detection of different rice traits

Rapid acquisition of physiological and biochemical phenotypic information of crops, such as pigment content, water content, and photosynthetic rate, is crucial for describing key crop traits and providing decision support for predicting crop yield and monitoring growth and stress response (Rebetzke et al. 2019; Yang et al. 2019a). In recent years, high-throughput phenotype technologies have emerged, employing spectral and imaging technology for the nondestructive acquisition of crop physiological and biochemical parameters, contributing significantly to the digitization and intelligent management of agriculture operations (Feng et al. 2020; Yang et al. 2020a). These technologies significantly contribute to achieving agricultural precision, digitization, information and intelligent management operations.

## ML- and DL-facilitated improvement in rice chlorophyll measurement

Chlorophyll is a crucial parameter in crop biology, providing insights into plant nutrient stress, pest and disease detection, and growth and senescence (Kalaji et al. 2016; Shah et al. 2017). Traditionally, the quantification of chlorophyll involves a chemical and destructive process, which is time-consuming and impractical in the field (Croft et al. 2017). However, chlorophyll content is closely related to rice photosynthetic capacity and growth, making accurate detection essential for monitoring vegetative growth and diagnosing fertilization (Evans and Clarke 2019). As an alternative, Stavrakoudis et al. (2019) introduced a vegetation index for rice chlorophyll using multispectral imaging, allowing for precise fertilization. Changes in chlorophyll content in rice leaves, due to pathogen infections, can also be assessed using non-invasive chlorophyll fluorescence measurements. Zhou et al. (2014) analyzed chlorophyll fluorescence spectra of rice leaves at different disease stages, providing valuable insights.

Furthermore, Zhu et al. (2019) conducted research using rice seedlings infected with *Phytophthora* blight, combining hyperspectral imaging technology to predict chlorophyll content regression in rice leaves under rice blight stress. This approach enables the early identification of rice sheath blight. Integrating spectral and fluorescence data facilitates the characterization of physiological and biochemical information in plants. Moreover, the intrinsic relationship between biochemical components within leaves and photosynthetic physiology enhances our understanding of responses to various diseases and their impact on rice production. This knowledge provides valuable insights and theoretical guidance for optimizing canopy photosynthesis and increasing crop yields.

## Other physiological and biochemical indicators

Other physiological and biochemical indicators have been studied for their potential to differentiate between susceptible and normal rice samples, based on ML and DL, contributing to early judgments of rice infections (Kutubuddin et al. 2020). However, few studies have been conducted on the physiological and biochemical indicators that undergo changes in rice specimens. Most studies have used infrared thermography (Gao et al. 2013), electron microscopy (Elshayb et al. 2022) and tunable diode laser absorption spectroscopy (Yang et al. 2020b) to detect changes in the internal material composition or physiological and biochemical status of the objects, enabling early diagnosis of minor changes brought about by diseases.

When rice crops are affected by disease stress, significant changes in photosynthesis and transpiration occur in the affected sites, leading to significant differences between infected and healthy leaves. Wang et al. (2019) summarized the progress in infrared thermal imaging technology combined with DL algorithms for early diagnosis of crop diseases. They highlighted how DL can detect diseases by recognizing infrared thermal images, improving the shortcomings of these images, and enhancing the speed and accuracy of disease identification. Hamada et al. (2020) emphasized the use of infrared thermal imaging to monitor abnormal temperature changes in infected areas, providing the basis for early disease diagnosis. Miyazaki et al. (2016) determined the complete viroid structure of rice dwarf virus (RDV) containing the P2 protein by cryo-electron microscopy, observing the partial structure of P2 and position in the capsid. They also examined the 3D structure of RDV at different stages of virus entry into cells by electron tomography, providing insights into the cellular attachment and entry of RDV. Further amalgamation of various techniques, particularly through computer technology, is anticipated to enhance these methods further and assume a more prominent role in plant disease research.

## ML and DL at the genomic scale

ML has gained an important role in genomics research following advancements in high-throughput data generation technologies (Marx 2013). This review focuses on how the genomics data can interact with the other types of data like chemical and images analyses (Dias and Torkamani 2019; Libbrecht and Noble 2015; Wu et al. 2014). ML identifies these interactions and extracts essential information from complex datasets (Jankovic and Gojobori 2022; Yip et al. 2013). This processing model algorithm has been successful in numerous large-scale data analysis domains, such as genomics, transcriptomics, proteomics, and systems biology, enabling the identification of promoters, enhancers, splicing patterns, transcription factors, and RNA-binding proteins. However, ML development has been predominantly in the biomedical field, with relatively few applications targeting plant or plant pathogen genomics.

Balancing the utilization of ML in genomics requires finding the right equilibrium between the model data and the training data. Simple models may struggle to describe data with highly complex distributions, necessitating harmony between the predicted and training models. A significant challenge in applying ML to genomics is the scarcity of real data, with the training dataset often vastly outnumbering the test dataset (Sperschneider 2020). Furthermore, genomic data can be high-dimensional, while the number of observations remains limited (Reel et al. 2021). For example, whole-genome sequencing data typically contain tens of thousands of genetic information, yet only a small fraction of observational data can be annotated.

**Fig. 3:**
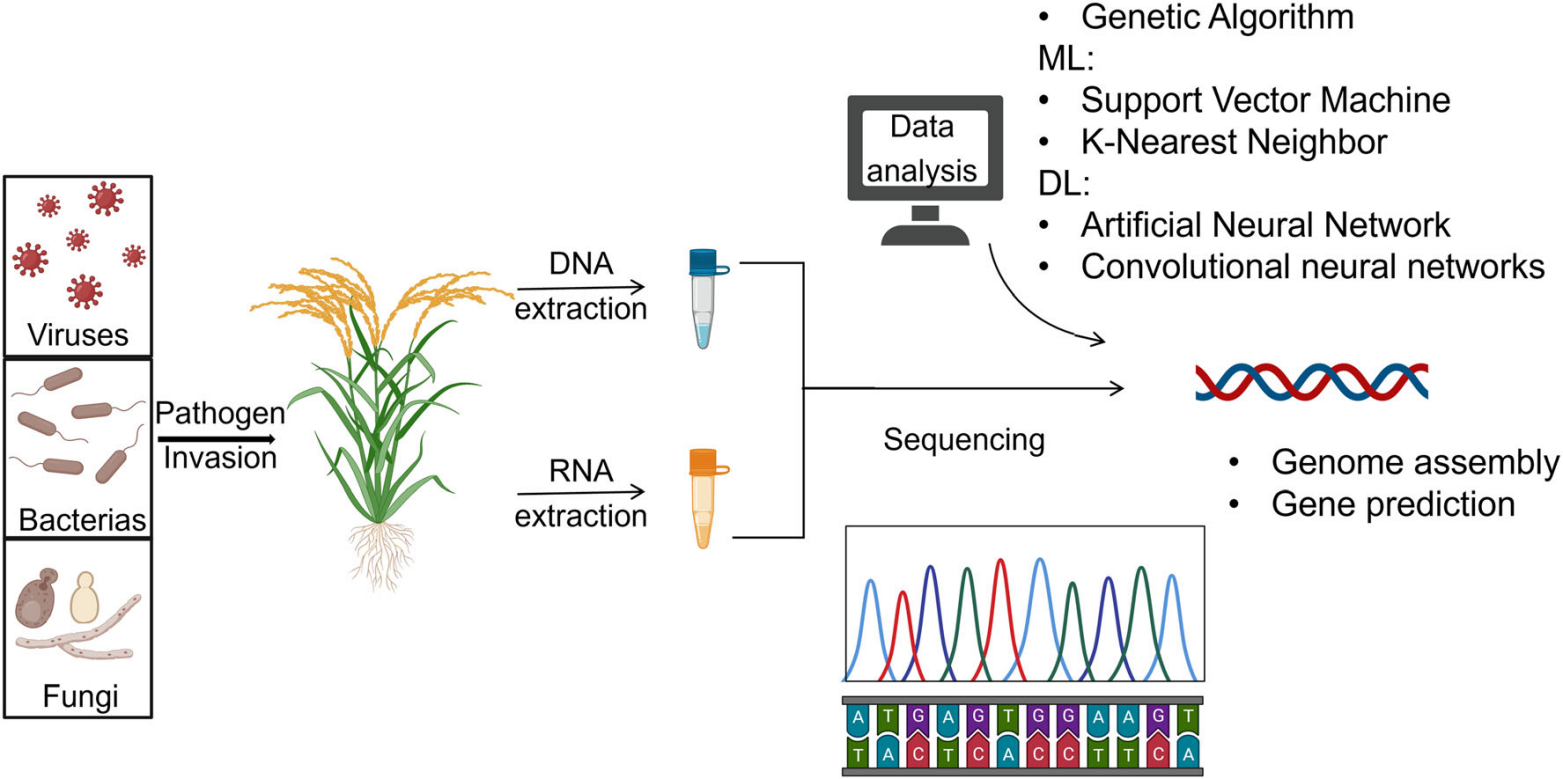
Application of ML and DL in plant–pathogen interactions (Created with BioRender.com)

In terms of plant–pathogen interactions from a genomic perspective, the principal application areas of ML encompass the prediction of gene regulatory networks, genomic selection for disease resistance, and the prediction of pathogen effector proteins (Singh et al. 2016; Sperschneider 2020) (Fig. 3). Large-scale plant-based transcriptome sequencing datasets can be leveraged to infer gene regulatory networks and identify genes involved in plant–pathogen interactions (Li et al. 2021; Yang et al. 2019b). For instance, Das et al. (2022) conducted a comparative time-series RNA-Seq analysis of a widely grown rice variety (BPT-5204) and employed an integrated ML and network-based approach to construct a rice transcriptional regulatory network (TRN) at three different time points. This approach enabled the identification of regulatory hubs critical to the early and late responses of rice to *Rhizoctonia solani*. Besides, Shaik and Ramakrishna (2014) used stress response genes to distinguish various stress conditions and identify candidate genes for extensive resistance in rice. SVM models were also utilized to classify stress responses as biotic or abiotic differentially expressed genes from 559 microarray samples under 13 stress conditions. Cernadas et al. (2014) applied Naive Bayes (NB) and Logistic Regression (LR) algorithms to discover the effector targets of the bacterial leaf streak pathogen, *Xanthomonas oryzae pv. Oryzicola* (Xoc), in rice, based on transcriptomics data.

Recently, DL has gained prominence in genomics due to its high learning capacity, wide applicability, and excellent portability (Eraslan et al. 2019). The primary areas of DL application in plant–pathogen interactions include gene expression regulation, anomaly detection, and functional genomics. Kumar et al. (2022) developed a DL-based rice network model (DLNet) to quantitatively explore differences and reveal distinct adaptive strategies rice plants employ to evade pathogen effectors. Although DL displays significant potential in this field, challenges remain, such as the mechanistic exploration of rice interactions with various pathogens, hindered by the lack of extensive datasets involving rice's interactions with different pathogens. However, these challenges could diminish with the accumulation of more data and advancements in DL.

It is widely thought that future advancements in dissecting plant–pathogen interactions will be driven by integrating diverse genomic data sources, with ML and DL playing pivotal roles in their advanced applications (Cheifet 2019; Mochida et al. 2018). As the quality of interactome datasets continues to improve, ML and DL are expected to find broader applications in genomics, serving as facilitating tools for analyzing plant–pathogen interaction data with high resolution.

## Conclusion and future perspectives

In summary and looking ahead, the application of rice disease detection technology in disease detection is a crucial area of research. The utilization and advancement of advanced detection technologies like hyperspectral imaging and infrared thermal imaging have made it possible to effectively monitor numerous early-stage diseases that were previously undetectable. The multidimensional sample data obtained from data cubes collected through hyperspectral images provide valuable insights. There is a growing trend in employing advanced model algorithms for rice disease detection and recognition. Combining these model algorithms ensures both the reliability of rice yield and quality and the timely and precise availability of disease-related information. This paper offers a comprehensive review of monitoring rice diseases using these advanced techniques across various scales. The application of emerging disease detection technology has the potential to significantly improve the identification rate of rice diseases, leading to precise solutions for crop growers and reduce economic losses related to disease detection. Furthermore, this research lays a solid foundation for the prompt detection of rice diseases and holds significant reference value in terms of safeguarding the economic losses associated with essential national food security crops, including rice. Additionally, this paper explores the application of high-throughput data from microbial genomes in conjunction with model algorithms for predicting rice diseases. Machine learning and deep learning have produced remarkable results in detecting and classifying high-throughput microbiome data (Zhan et al. 2022). Leveraging advanced model algorithms allows valuable information within microbial genomes to be efficiently extracted and translated into phenotype links, enabling experimental modeling and multi-scale computational simulations using biometric approaches. As a result, integrating microbiome and artificial intelligence technology promises even greater potential in future.

In future crop disease detection research, several key considerations are expected. First, there is a pressing need to address the limited transferability of specific model algorithms. For example, deep neural networks display varying capabilities to transfer abstract features learned at different layers. Shallow layers tend to exhibit relatively stronger transferability compared to deeper layers. However, as the network's depth increases, the learned features become more specialized and lose their transferability. Additionally, in practical applications, it is often of utmost importance to scrutinize why the model predicts a particular data point to have a specific value, a concept known as local interpretability. Random forest models inherently possess this local interpretability, as one can trace the decision path through the branches to comprehend the prediction. On the other hand, achieving such local interpretability represents a formidable challenge for deep learning models. While running data through the model and observing activated neurons can offer some insights, the interpretation of individual neurons or neuron clusters remains uncertain. Even minor alterations in a feature's value in the data frequently lead to substantially different model predictions. This situation renders the application of counterfactual analysis, which entails studying how changes in input data affect model predictions, impractical for achieving local explainability. Therefore, future efforts should focus on enhancing the stability and interpretability of various models. Moreover, optimizing and amalgamating different models can contribute to the more precise detection of rice diseases across diverse regions and categories.

## Data Availability

Data sharing is not applicable to this article as no datasets were generated or analyzed during the current study.
